# Direction of TDCS current flow in human sensorimotor cortex influences behavioural learning

**DOI:** 10.1016/j.brs.2019.01.016

**Published:** 2019

**Authors:** Ricci Hannah, Anna Iacovou, John C. Rothwell

**Affiliations:** Sobell Department of Motor Neuroscience and Movement Disorders, UCL Institute of Neurology, London, UK

**Keywords:** Transcranial magnetic stimulation, Transcranial direct current stimulation, Motor cortex, Learning, Plasticity, TMS, Transcranial magnetic stimulation, TDCS, transcranial direct current stimulation, AP, anterior-posterior, PA, posterior-anterior, MEP, motor evoked potential, APB, abductor pollicis brevis, ADM, abductor digiti minimi

## Abstract

**Background:**

Recent studies have shown that neurophysiological outcomes of transcranial direct current stimulation (TDCS) are influenced by current flow in brain regions between the electrodes, and in particular the orientation of current flow relative to the cortical surface.

**Objective:**

We asked whether the directional effects of TDCS on physiological measures in the motor system would also be observed on motor behaviours.

**Methods:**

We applied TDCS during the practice of a ballistic movement task to test whether it affected learning or the retention of learning 48 h later. TDCS electrodes were oriented perpendicular to the central sulcus and two current orientations were used (posterior-anterior, TDCS_PA_; and anterior-posterior, TDCS_AP_). Transcranial magnetic stimulation (TMS) was used to assess whether changes in corticospinal excitability reflected any behavioural changes.

**Results:**

Directional TDCS_AP_ impaired the retention of learning on the ballistic movement task compared to TDCS_PA_ and a sham condition. Although TDCS_PA_ had no effect on learning or retention, it blocked the typical increase in corticospinal excitability after a period of motor practice.

**Conclusions:**

Our results extend on previous reports of TDCS producing directionally specific changes in neurophysiological outcomes by showing that current direction through a cortical target also impacts upon behavioural outcomes. In addition, changes in corticospinal excitability after a period of motor practice are not causally linked to behavioural learning.

## Highlights

• TDCS current direction influences neurophysiological outcomes.
• We show that it also influences behavioural outcomes.
• Behavioural learning is not linked to changes in corticospinal excitability.

## Introduction

Methods of transcranial electrical stimulation of the brain, particularly transcranial direct current stimulation (TDCS), which is low cost and easy to apply, have achieved widespread popularity. Yet there is growing disquiet about the replicability and effect size of many of the results that have been reported (e.g. Refs. [[1], [2], [3]]). Many factors can contribute to this, such as variation in levels of current that reach the cortex and levels of activity in the brain at the time of stimulation [[4], [5], [6], [7], [8], [9]]. One factor that we have studied recently is the effect of applied field direction with respect to the orientation of cortical neurones [10,11]. Although the influence of TDCS on the membrane potential of a flat sheet of perpendicularly oriented neurones is easy to calculate, it is much more difficult to predict the overall effect of the same current on the folded human cortex.

**Fig. 1:**
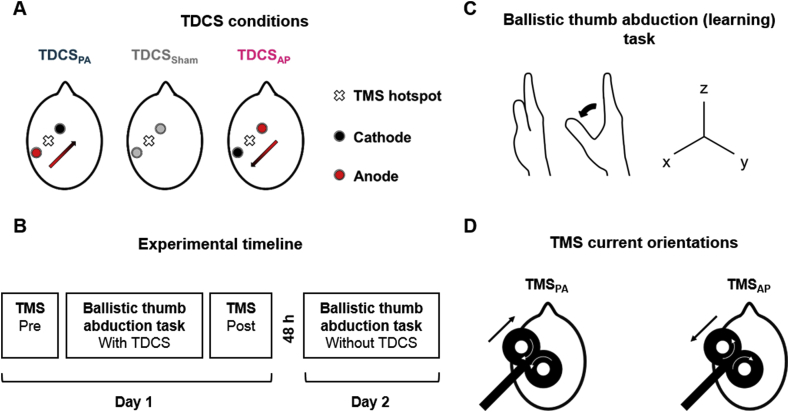
***A*.** Three TDCS conditions: anode posterior and cathode anterior to TMS hotspot (posterior-anterior current, TDCS_PA_), anode anterior and cathode posterior (anterior-posterior current, TDCS_AP_), and sham (TDCS_Sham_). ***B*.** Protocol schematic depicting timeline of events. ***C***. Task used to assess learning involved performing ballistic abduction movements of the thumb where the aim was to increase the acceleration in the x-axis (abduction) over time with practice. ***D*.** MEPs were evoked in hand muscles by applying using TMS over the hotspot. Black arrows the direction of current induced in the brain (PA and AP) and white arrows indicate the direction of current in the coil.

The importance of the orientation of the applied field was noted in the first study of Nitsche and Paulus (2000). They found that TDCS only led to after-effects on motor cortex excitability if one electrode was over the hand area of the primary motor cortex (M1) and the other over the contralateral orbit. We recently confirmed this by showing that TDCS applied so as to produce a field oriented in a posterior-anterior direction (TDCS_PA_, Fig. 1A), perpendicular to the line of the central sulcus, suppressed cortical excitability whereas a latero-medial field had no effect [10]. Field orientation can also influence the amount of motor cortex plasticity produced by concurrent repetitive transcranial magnetic stimulation (rTMS) [11]. Thus, TDCS_PA_ tends to increase the facilitatory after-effect of intermittent theta burst stimulation (iTBS) applied with a monophasic posterior-anterior oriented TMS current (TMS_PA_), whilst anterior-posterior TDCS (TDCS_AP_) has the opposite effect. The present experiments were designed to test whether the directional effects of TDCS on physiological measures would also be observed on motor behaviours.

We chose a ballistic thumb acceleration task since previous work with TMS had found evidence that learning could be improved by prior conditioning of neural elements sensitive to TMS_PA_, but not those sensitive to AP TMS currents (TMS_AP_) [13]. We predicted, in light of the effects of TDCS on concurrent iTBS [11], that TDCS_PA_ might improve learning, whereas TDCS_AP_ would impair it. We made no predictions about how the improvement/worsening of learning might take place since previous work with non-directional TDCS had shown it might improve either online learning during the task (as in a serial reaction time or ballistic acceleration task) [6,14], or consolidation/retention following the task (in a speed-accuracy task or ballistic acceleration task) [[15], [16], [17]]. Therefore, we tested both.

Behavioural motor learning is also associated with an increase in corticospinal excitability to the trained muscles [[19], [20], [21]], which is suggested to reflect learning-related changes in motor cortical organisation. We recently suggested that a particular set of synaptic inputs to the corticospinal neurones, which can be probed with TMS_PA_ (PA-sensitive neurones), were specifically involved in learning of a ballistic motor task, whilst another set (AP-sensitive neurones) were not [13]. This might mean than only changes in specific circuits are relevant to behavioural learning. We therefore expected that the changes in corticospinal excitability would follow those of motor learning, but that this would be specific to motor potentials evoked by TMS_PA_ and not those evoked by AP-directed TMS (TMS_AP_).

## Methods

### Participants

48 volunteers (25 females; mean age 23 years [range 19–34 years], 46 right-handed), who reported no contraindications to TMS [22], provided written informed consent prior to participating in the study which was approved by University College London Ethics Committee. On days where they attended the laboratory, participants were asked to avoid consuming caffeine and extensive motor practice (e.g. playing musical instruments and electronic games prior to the experiment).

### Experimental design

Participants visited the laboratory on two separate occasions separated by 48 h (Fig. 1B) to perform a ballistic thumb abduction task (Fig. 1C) where the aim was to increase the acceleration of the thumb throughout practice. They were randomly assigned to one of three TDCS groups (*N* = 16 per group): two active conditions involving opposite current directions and one sham (Fig. 1A). Both the experimenter (A.I.) and participants were blinded to the group allocation. On the day of testing, another researcher not involved in the data collection or analysis (R.H.) selected the stimulation condition and applied the stimulation. Unblinding was performed following the completion of data collection and analysis. The rationale for a between-subjects, rather than a within-subjects, design was to avoid order effects associated with incomplete wash-out of learning after the first experimental session (e.g. Rroji et al., 2015). On the first day, TDCS was applied across the sensorimotor cortex whilst participants performed the task, and on the second day the task was repeated without TDCS. Learning was assessed as the increase in peak abduction acceleration from the first to last block of practice on days 1 and 2, whilst retention of learning was assessed as the change in acceleration performance over a 48-h period, i.e. from the end of day one to the start of day 2. In order to assess changes in corticospinal excitability associated with learning on day 1, TMS was used to evoke MEPs from an agonist muscle involved in the task (abductor pollicis brevis, APB) and an uninvolved control muscle (abductor digiti minimi, ADM) immediately prior to and 5 min after completing the task. MEPs were evoked using two TMS current orientations (Fig. 1D) to examine changes in PA and AP sensitive synaptic inputs to the corticospinal neurones since they have been suggested to play different roles in motor preparation and learning [13,23].

### Ballistic thumb acceleration task

The task was similar to previous studies [13,17,24]. Participants were seated with their dominant arm slightly abducted, with the elbow flexed to ∼45° (where 0° is full extension) and the forearm semi-pronated. The wrist, hand and digits were secured in a custom-built rig to prevent movement except of the thumb. Participants performed ballistic thumb abduction movements of the dominant hand at a rate of 0.33 Hz indicated by a brief auditory tone (1000 Hz). On each day, they performed an initial baseline block of 20 trials. This was followed by two blocks of 120 trials separated by 4 min of rest in order to minimise fatigue.

The acceleration of the thumb was measured in the x, y and z planes using a tri-axial accelerometer (ACL300; Biometrics Ltd., UK) attached to the proximal phalanx of the thumb and aligned so that the abduction-adduction axis was equivalent to the x-axis of the accelerometer. The raw signal was amplified (1 mV/g; DataLOG; Biometrics Ltd., UK), digitised (1000 Hz; CED 1401; Cambridge Electronic Design, UK), smoothed (5 ms time constant), and stored on a personal computer for offline analysis. Participants received online visual feedback of the peak acceleration in the x-axis for each trial, as well as the greatest acceleration achieved within either of the two blocks, and were encouraged by the experimenter to improve upon their best performance throughout the training.

### Surface electromyogram (EMG)

Surface EMG electrodes (WhiteSensor 40713, Ambu^®^, Denmark) were placed in a belly-tendon arrangement over the APB and ADM muscles of the dominant hand. The ground electrode was placed over the styloid process of the radius. Signals were amplified with a gain of 1000 (Digitimer, UK), band-pass filtered (5–3000 Hz), digitised at 5000 Hz (1401; CED, Cambridge, UK), and analysed with Signal v5.10 software.

### Transcranial magnetic stimulation (TMS)

Single pulse TMS was used to evoke MEPs in the APB and ADM muscles via a MagPro X100 stimulator (MagVenture A/S, Denmark) connected to a figure-of-8 coil (MagPro MC-B70 Butterfly coil; MagVenture A/S, Denmark). The coil was held with the handle approximately perpendicular to the presumed orientation of the hand representation in the anterior bank of the central sulcus (∼45° from the sagittal plane). Monophasic pulses were applied to induce PA and AP currents in the brain using the in-built function on the device to select the direction of current in the coil. A neuronavigation system (Brainsight; Rogue Research Inc., Montreal, Quebec, Canada) was used to monitor and maintain coil positioning throughout the session.

The motor hot spot was initially found by searching for the position where slightly supra-threshold TMS_PA_ currents produced the largest and most consistent MEPs in APB. The position and orientation of the coil was logged in Brainsight and marked directly on the scalp using coloured pencil. This enabled precise positioning and orienting of TDCS electrodes (see below).

For each current direction, resting motor threshold (RMT) was defined as the intensity, in % of maximum stimulator output (MSO), required to produce a MEP in the APB muscle greater than 0.05 mV in 5/10 consecutive stimuli. Similarly, the test stimulus (TS) intensity was defined as that required to produce a MEP in the APB muscle greater than 0.5 mV in 5/10 consecutive stimuli. Thirty MEPs were evoked using the TS intensity for each current direction prior to and following motor practice, and the order of current directions was randomised across participants.

### Transcranial direct current stimulation (TDCS)

TDCS was applied using a StarStim device (Neuroelectrics, Spain). Two Ag/AgCl gelled electrodes (Pistim, 3.14 cm^2^; Neuroelectrics, Spain) were positioned 3.5 cm anterior and posterior to the TMS hotspot, and oriented parallel to the TMS coil orientation (i.e. perpendicular to the presumed orientation of the anterior bank of the central sulcus) (Fig. 1A; see also Tremblay et al., 2017; Rawji et al., 2018).

A current of 1 mA was applied for a total of 12 min whilst participants completed the motor practice on day 1, i.e. two blocks of 6 min separated by 4 min rest without stimulation. TDCS_Sham_ involved a ramping up and down of stimulation to 1 mA over 20 s at the start of each 6-min period.

### Data analyses

**Fig. 2:**
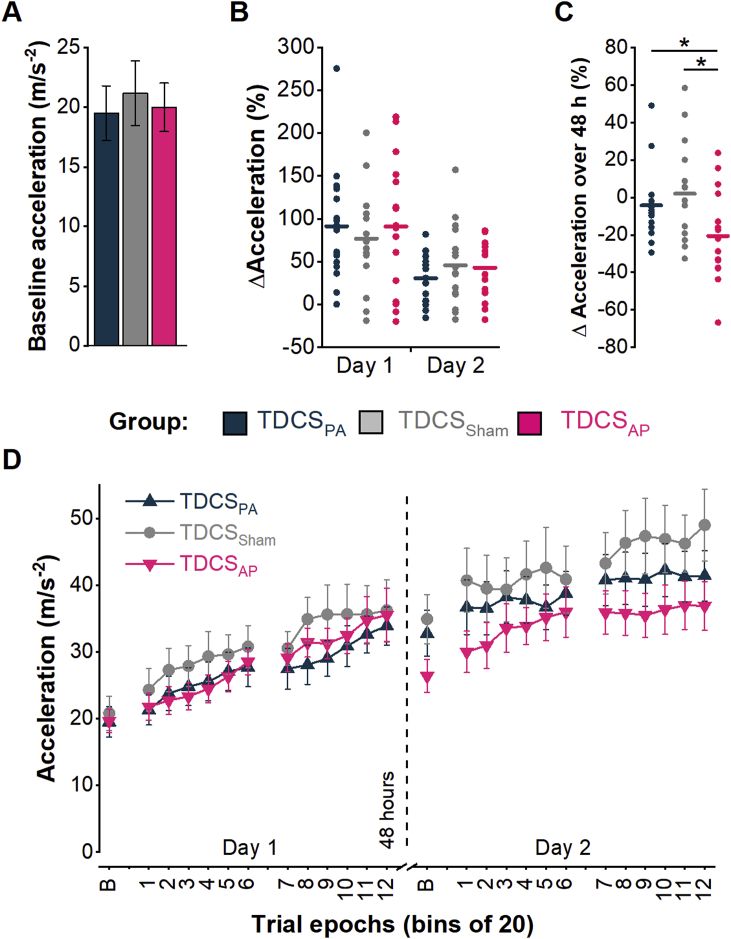
Performance during the ballistic thumb acceleration task. **A**. Absolute peak acceleration in the baseline epoch on day 1 was similar across the three TDCS groups. **B**. The relative increase in acceleration on day 1 and day 2 was similar across the three TDCS groups, but greater on day 1 than day 2. Circles reflect individual data points whilst solid lines represent the group means. **C**. The group with TDCS applied in with an AP current direction experienced a reduction in peak acceleration from the last epoch of day 1 to the baseline epoch of day 2 by comparison with the groups receiving PA and Sham TDCS. As in B, the circles reflect individual data points whilst solid lines represent the group means. **D.** Group mean acceleration for each epoch of practice across the 2 days. *P < 0.05 between groups.

**Fig. 3:**
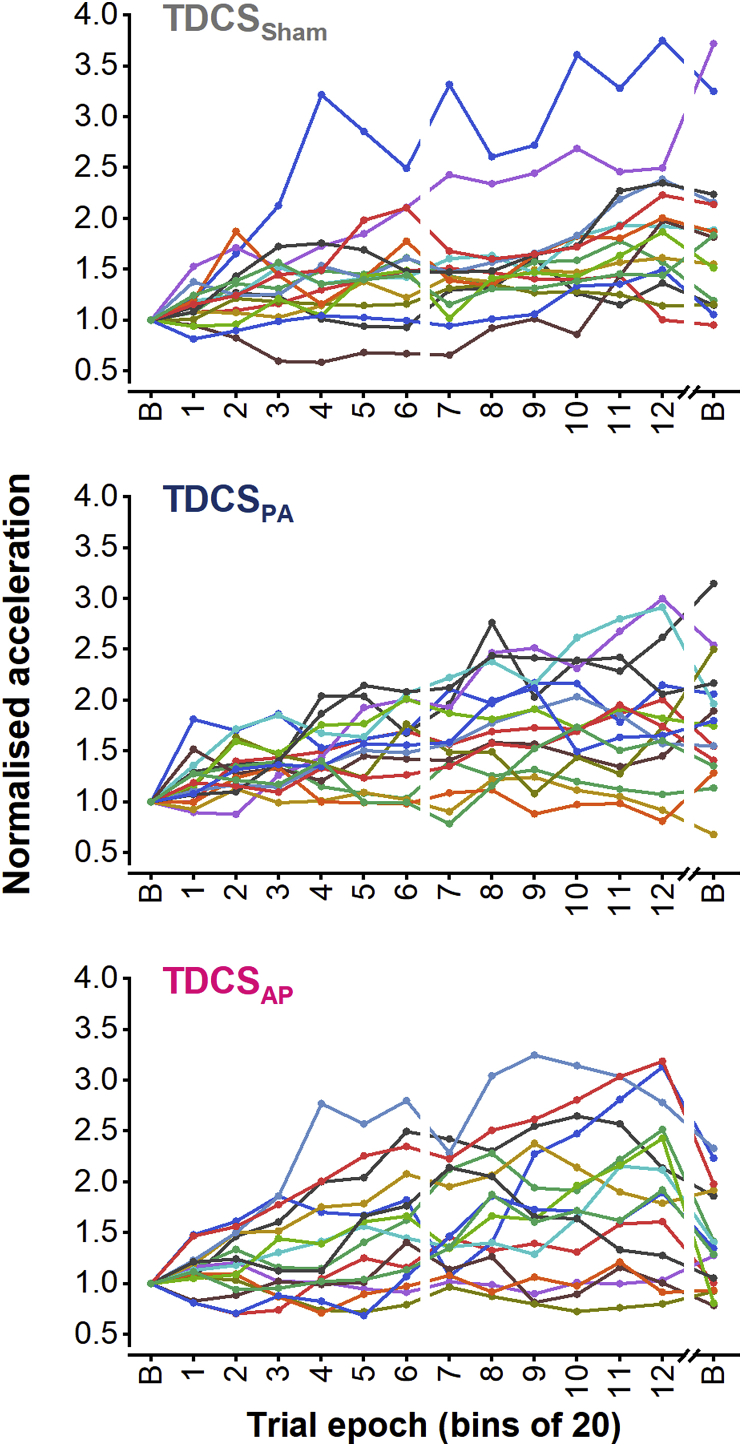
Individual data showing the acceleration for each epoch of practice across day 1 and the first block of practice on day 2, all normalised to the baseline epoch on day 1. Data are organised according to TDCS group (top, TDCS_Sham_; middle, TDCS_PA_; bottom, TDCS_AP_). Break in *x*-axis separates data from different days.

On each day, peak abduction acceleration (measured in the x-axis) was averaged across 20 consecutive trials to create 13 epochs: a baseline epoch and a further 12 epochs (2 blocks of 6). Learning on each day was quantified as the percentage change in acceleration from the baseline to the final epoch. Retention of learning was quantified as the percentage change in acceleration, from the final epoch on day 1 to the baseline epoch on day 2, where positive values indicate an increase in performance and negative values a decrease in performance over the 48 h. The group mean acceleration in every epoch, illustrating the full time course of changes over the two days, is shown in Fig. 2D and individual data (normalised to the acceleration in the baseline epoch of the first day by expressing as a ratio) are shown in Fig. 3.

Peak-to-peak MEP amplitudes in the APB and ADM evoked by TMS_PA_ and TMS_AP_ were measured on a trial-to-trial basis and used to calculate a mean value at each time pre- and post-motor practice. For each current direction and muscle, the post-practice MEP amplitudes were also expressed as a percentage change from the pre-practice MEP amplitudes.

### Statistical analyses

Analyses of the data primarily involved with ANOVA with a between-subjects factor of TDCS group (TDCS_PA_, TDCS_AP_ and TDCS_Sham_). For the analysis of learning, we first confirmed that there were no differences in the absolute baseline performance by entering the data from the baseline block on day 1 into an ANOVA with TDCS group (TDCS_PA_, TDCS_AP_ and TDCS_Sham_) as a between-subjects factor. Then, the percentage changes in acceleration on each day were entered into an ANOVA with day as a within-subjects factor (day 1 and day 2) and TDCS group as a between-subjects factor (TDCS_PA_, TDCS_AP_ and TDCS_Sham_). The retention of learning involved entering the percentage change in acceleration (i.e. from the final epoch on day 1 to the baseline epoch on day 2) into an ANOVA with TDCS group as a between-subjects factor (TDCS_PA_, TDCS_AP_ and TDCS_Sham_). The rationale for analysing learning and retention separately is that they appear to be relatively separate processes, for example, conventional anodal TDCS [16,17] and repetitive TMS [26] have been shown to modulate the consolidation of learning without affecting the rate of learning itself.

TMS data were analysed by first confirming that TS intensities and absolute MEP amplitudes at baseline were similar between the groups. TS intensities were entered into an ANOVA with TMS current direction as a within-subjects factor (TMS_PA_ and TMS_AP_) and TDCS group as a between-subject factor (TDCS_PA_, TDCS_AP_ and TDCS_Sham_). Absolute MEP amplitudes prior to motor practice were entered into an ANOVA with within-subjects factors of TMS current direction (TMS_PA_ and TMS_AP_) and muscle (APB, ADM), and a between-subjects factor of TDCS group (TDCS_PA_, TDCS_AP_ and TDCS_Sham_).

Subsequently, an omnibus ANOVA was performed on the absolute amplitudes with within-subjects factors of TMS current direction (TMS_PA_ and TMS_AP_), muscle (APB, ADM) and time (pre- and post-practice), and a between-subjects factor of TDCS group (TDCS_PA_, TDCS_AP_ and TDCS_Sham_). Follow-up analyses involved the percentage change in MEP amplitudes being entered into separate ANOVAs for the APB and ADM muscles, each with current direction as a within-subjects factor (TMS_PA_ and TMS_AP_) and TDCS group as a between-subjects factor (TDCS_PA_, TDCS_AP_ and TDCS_Sham_).

The relationships between the percentage change in MEP amplitude and learning, as well as changes in MEP amplitude and retention, were assessed using Pearson's correlations applied within each TDCS group and across the whole cohort.

Significant main effects were followed up by independent samples *t*-tests. Data are presented as group mean ± SEM. Where necessary, the Greenhouse-Geisser procedure was applied to correct for violations of sphericity in ANOVA. *P* values < 0.05 were considered significant. Effect sizes are reported as partial eta squared (*η*p^*2*^), where 0.01, 0.06 and 0.14 are considered small, medium and large effect sizes.

## Results

### Motor learning and retention

There was no difference between the groups in absolute baseline acceleration (one-way ANOVA: F_[2,45]_ = 0.105, *P* = 0.900, *η*p^*2*^ = 0.005; Fig. 2A). Analysis of the relative change in acceleration showed that there were no differences between the groups in the practice-related improvement across the two days, though overall there was less improvement on day 2 than day 1 [two-way ANOVA: day (F_[1,45]_ = 17.912, P < 0.001, *η*p^*2*^ = 0.285), TDCS group (F_[2,45]_ = 0.068, P = 0.934, *η*p^*2*^ = 0.003), TDCS group × day (F_[2,45]_ = 0.436, P = 0.649, *η*p^*2*^ = 0.019; Fig. 2B].

One-way ANOVA on the retention of learning showed an effect of TDCS group (F_[2,45]_ = 4.134, P = 0.022; *η*p^*2*^ = 0.155; Fig. 2C). *Post hoc t*-tests revealed a greater reduction in acceleration over the 48 h period for TDCS_AP_ compared to TDCS_PA_ (P = 0.039) and TDCS_Sham_ (P = 0.015), with no difference between the latter two groups (P = 0.44). Thus, TDCS_AP_ impaired the retention of learning compared to TDCS_PA_ and TDCS_Sham_.

### Corticospinal excitability

Baseline TMS intensities and MEP amplitudes measured prior to practice were similar across the three TDCS groups (Table 1). ANOVA revealed that RMT and TS intensities were significantly greater for AP compared to PA currents, as expected [27,28], but there were no differences between the three TDCS groups (Table 2). ANOVA on absolute MEP amplitudes prior to practice showed no main effect or interactions, confirming similar MEP amplitudes across TDCS groups, muscles and TMS current directions (Table 2).

**Table 1:** TMS thresholds and baseline MEP amplitudes measured with each current direction.

	RMT (%MSO)		TS intensity (%MSO)		Pre-practice MEP amplitude (mV)			
	TMS_PA_	TMS_AP_	TMS_PA_	TMS_AP_	TMS_PA_		TMS_AP_	
	APB	APB	APB	APB	APB	ADM	APB	ADM
TDCS_PA_	51 ± 2	68 ± 4	57 ± 3	77 ± 4	0.69 ± 0.06	0.62 ± 0.06	0.59 ± 0.15	0.48 ± 0.14
TDCS_Sham_	56 ± 2	76 ± 3	67 ± 2	89 ± 3	0.58 ± 0.04	0.58 ± 0.07	0.62 ± 0.18	0.63 ± 0.15
TDCS_AP_	55 ± 4	69 ± 3	64 ± 4	83 ± 4	0.64 ± 0.04	0.60 ± 0.05	0.54 ± 0.18	0.52 ± 0.15

**Table 2:** Results of ANOVAs performed on motor thresholds and pre-practice MEP amplitudes.

	RMT			TS intensity			MEP amplitude		
	*F*_[DF,error]_	P	*η*p^*2*^	*F*_[DF,error]_	P	*η*p^*2*^	*F*_[DF,error]_	P	*η*p^*2*^
TDCS group	1.336_[2,45]_	0.273	0.056	3.015_[2,45]_	0.059	0.118	0.035_[2,45]_	0.996	0.002
TMS current direction	**294.1**_[**1,45**]_	< **0.001**	**0.867**	**374.1**_[**1,45**]_	< **0.001**	**0.893**	0.416_[1,45]_	0.502	0.009
TDCS group × TMS current direction	2.446_[2,45]_	0.098	0.098	0.360_[2,45]_	0.700	0.016	0.214_[1,45]_	0.808	0.009
Muscle	–	–	–	–	–	–	0.502_[1,45]_	0.482	0.011
TDCS group × Muscle	–	–	–	–	–	–	0.452_[2,45]_	0.639	0.020
Muscle × TMS current direction	–	–	–	–	–	–	0.003_[1,45]_	0.954	0.000
TDCS group × TMS current direction × Muscle	–	–	–	–	–	–	0.037_[2,45]_	0.964	0.002

**Fig. 4:**
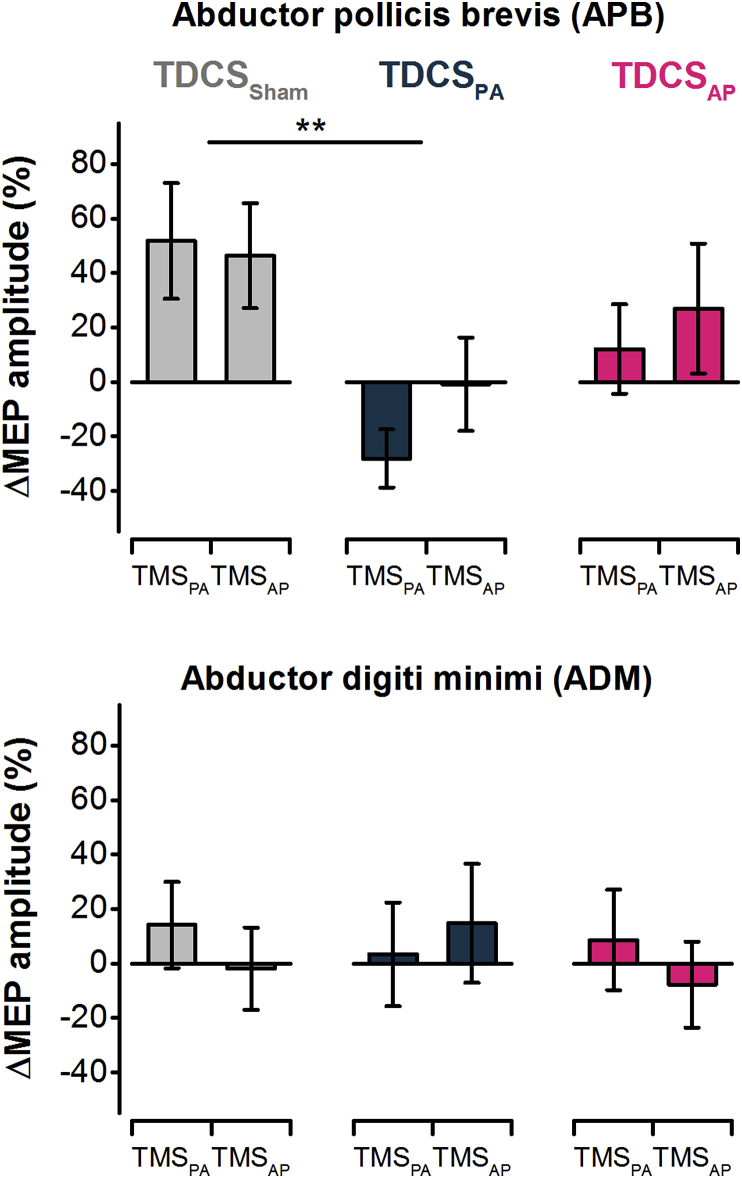
Percentage change in MEP amplitude following practice on the ballistic thumb acceleration task combined with online TDCS. Application of TDCS in a PA current direction reversed the normal effect of practice (i.e. sham TDCS) on MEPs in the agonist muscle (abductor pollicis brevis). TDCS applied in the opposite AP current direction had no overall effect. Overall, changes in MEPs were similar when evoked with PA and AP TMS currents. MEPs in uninvolved abductor digiti minimi muscle were unaffected by motor practice and TDCS. **P < 0.01.

The omnibus ANOVA showed a significant interaction of TDCS group × Muscle × Time (Table 3). Separate follow-up ANOVAs for the APB and ADM muscles showed a significant interaction of TDCS group × Time for the APB muscle only (Table 3). One-way ANOVA was performed on the percentage change in APB MEP amplitudes with TDCS group as the between-subjects factor. Since there were no main effects or interactions with TMS current direction, the data were collapsed across TMS_PA_ and TMS_AP_. There was a main effect of TDCS group (F_[2,45]_ = 4.779, *P* = 0.013, *η*p^*2*^ = 0.175), with *post hoc* tests confirming a greater increase in MEP amplitudes for TDCS_Sham_ compared to the TDCS_PA_ (*P* = 0.006), with no differences between TDCS_PA_ and TDCS_AP_ groups (*P* = 0.210) or between TDCS_Sham_ and TDCS_AP_ groups (*P* = 0.589). To summarise, in the absence of real TDCS (i.e. TDCS_Sham_), MEP amplitudes increased in the APB and were unchanged in the ADM after motor practice (Fig. 4), as would be expected [19]. However, *verum* TDCS affected the change in APB MEP amplitudes in current direction-dependent manner, whereby TDCS_PA_ appeared to suppress MEP amplitudes whereas the effects of TDCS_AP_ were less clear since the changes fell in between those of TDCS_Sham_ and TDCS_PA_. Additionally, the changes in MEP size across all three TDCS conditions appeared to be similar for both TMS current directions.

**Table 3:** Results of ANOVAs performed on absolute MEP amplitude pre- and post-practice.

	Omnibus ANOVA			APB muscle			ADM muscle		
	*F*_[DF,error]_	P	*η*p^*2*^	*F*_[DF,error]_	P	*η*p^*2*^	*F*_[DF,error]_	P	*η*p^*2*^
TDCS group	0.435_[2,45]_	0.650	0.028	0.730_[2,45]_	0.488	0.05	0.198_[2,45]_	0.821	0.009
Muscle	3.661_[1,45]_	0.062	0.090	–	–	–	–	–	–
TMS current direction	0.362_[1,45]_	0.550	0.008	0.215_[1,45]_	0.645	0.004	1.150_[1,45]_	0.298	0.025
Time	0.328_[1,45]_	0.569	0.018	**4.554**_[**1,45**]_	**0.038**	**0.099**	1.461_[1,45]_	0.233	0.031
TDCS group × Muscle	0.085_[2,45]_	0.918	0.004	–	–	–	–	–	–
TDCS group × TMS current direction	0.053_[2,45]_	0.949	0.002	0.386_[2,45]_	0.682	0.018	0.076_[2,45]_	0.927	0.003
TDCS group × Time	2.110_[2,45]_	0.133	0.018	**5.291**_[**2,45**]_	**0.009**	**0.187**	0.009_[2,45]_	0.992	0.000
Muscle × TMS current direction	2.008_[1.45]_	0.163	0.041	–	–	–	–	–	–
Muscle × Time	**8.495**_[**1,45**]_	**0.006**	**0.158**	–	–	–	–	–	–
TMS current direction × Time	0.032_[1,45]_	0.859	0.000	3.536_[1,45]_	0.067	0.069	1.371_[1,45]_	0.248	0.03
TDCS group × Muscle × TMS current direction	0.390_[2,45]_	0.679	0.017	–	–	–	–	–	–
TDCS group × Muscle × Time	**3.958**_[**2,34**]_	**0.026**	**0.155**	–	–	–	–	–	–
TDCS group × TMS current direction × Time	1.600_[2,45]_	0.213	0.069	2.440_[2,45]_	0.099	0.103	0.516_[2,45]_	0.600	0.022
Muscle × TMS direction × Time	**6.032**_[**1,45**]_	**0.018**	**0.116**	–	–	–	–	–	–
TDCS group × Muscle × TMS current direction × Time	0.522_[2,45]_	0.597	0.024	–	–	–	–	–	–

Finally, we found no correlation between the change in MEP amplitudes (TMS_PA_ or TMS_AP_) with the amount of learning on day 1 or the retention of learning over 48 h, and this was true both when assessed within each TDCS group (all *P* ≥ 0.116, *N* = 16 per group) and across the whole cohort (all *P* ≥ 0.173, *N* = 48).

### Exploratory analyses of movement kinematics

Given the seemingly divergent impact of TDCS on MEPs and retention of learning, we performed further exploratory analyses of movement kinematics to identify whether TDCS affected any movement parameters during learning in a way that could explain why TDCS_AP_ impaired retention (see Supplementary Fig. 1). The results showed the net direction of the thumb movement systematically changed throughout learning, implying that part of improvement in performance involves working out the right pattern of inputs to the target agonist and synergist muscles, whilst minimizing input to any antagonist muscles, in order to produce movements in the right direction. However, TDCS had no effect on the change in movement direction during learning.

### Relationship between the attainment of asymptotic levels of performance and the subsequent retention of learning

Previous research had indicated that, when learning a novel task, practicing at asymptotic levels of performance may help to stabilise newly formed motor memories and enhance retention [29]. Moreover, M1 seems to play a critical role in the process. In the present study, TDCS_AP_ could potentially have affected retention by influencing the rate of learning, and therefore the time spent practicing at asymptotic levels of performance. We therefore investigated the relationship between the slope of learning in the second block of practice on day 1 (whereby a lower slope would reflect nearer to asymptotic performance) and retention, and whether any effects of TDCS on retention were additive to or independent of the influence of slope (Supplementary Fig. 2). The results showed that TDCS group did not affect the rate of learning. Both TDCS and the slope of learning affected retention, but their contributions were independent.

## Discussion

The present results show that directional TDCS_AP_ impairs retention of learning in a ballistic movement task, whereas TDCS_PA_ has no effect. The implication is that some neural elements involved in retention align in the PA/AP direction across the central sulcus, and could perhaps be located in the posterior or anterior bank. The effect of TDCS_AP_ is in line with its reduction of the excitatory after-effects of iTBS_PA_ [11]. In those experiments, TDCS_PA_ only had a (non-significant) tendency to increase the response to iTBS_PA_, which may account for the lack of influence on behaviour in the present experiment. This could reflect some “ceiling” effect of directional TDCS. Unexpectedly, although TDCS_PA_ had no effect on learning it abolished the post-practice increase in MEPs, indicating that MEP changes have no direct relationship to learning. We conclude first that the direction of the applied electric field produced by TDCS determines its behavioural effects; and second, changes in corticospinal excitability observed after a period of movement practice are mechanistically unrelated to behavioural learning.

### Directional TDCS affects the retention of learning, but not learning, of a motor task

We recently showed that direction of current flow through a cortical target plays an important role in determining the neurophysiological outcomes of TDCS [10,11]. The present experiment examined whether the direction of current flow also determines its behavioural consequences. The data were consistent with this idea: the retention of learning on a ballistic motor task appeared to be affected by AP, but not PA, directed current flow. Though speculative, this lends to the prediction that one source of variability in TDCS outcomes [1,2,30] arises from poor control of current direction relative to the cortical target, and is determined by the interaction of the electrode configuration with individual anatomic factors [7,9].

From a mechanistic point of view, we know that TDCS currents will preferentially polarise neural elements that are aligned with the direction of current flow [[31], [32], [33]]. Therefore, some of the neural elements involved in the retention of the new motor memories were presumably aligned with the direction of the applied electric field. Current flow models of the present electrode montage predict uniformly oriented flow perpendicular to the anterior and posterior walls of the central sulcus [10]. In the neocortex, pyramidal cells are aligned perpendicular to the cortical surface whilst cortical interneurons and projection neurones tend to be aligned parallel to the cortical surface. The effects of TDCS_AP_ on retention could therefore be due to changes affecting pyramidal neurones in those areas (i.e. M1 or primary somatosensory cortex), or interneurons or projection neurones up in the pre-/post-central gyri.

The finding that TDCS_AP_ affected retention, whilst TDCS_PA_ did not, is difficult to explain. Traditional explanations for the polarity-dependent effects of TDCS [12] suggested that the soma and dendrites would be oppositely polarised according the direction of the field. The net depolarisation/hyperpolarisation of the soma would determine changes in neural activity and plasticity, and in turn dictate any behavioural outcomes. A prediction of this is different field directions (PA/AP) should produce opposite outcomes, but we did not find this to be the case. Such simplistic predictions have been criticised because the effects of TDCS may not simply reflect changes at the soma, but also those at the dendrites and pre-/post-synaptic terminals [8,31,32]. For example, data from hippocampal slices show that different orientations of DCS may modulate synaptic efficacy at different sites on the same neurones (e.g. different parts of the dendritic tree) [8], and therefore could affect different input pathways to the same post-synaptic neurones. Moreover, in the present study, the relative direction of the current with respect to the cortical surface would be opposite in the anterior and posterior walls of the sulcus, and could lend to differential changes in the motor and somatosensory cortices, the net effects of which are difficult to predict.

### A possible role for electrode location

Previous studies of the effects of TDCS on motor learning used an M1-orbitofrontal electrode montage and reported both online and offline effects. One reported an enhancement of learning in a ballistic movement task [6]. A more consistent finding is an offline improvement in the consolidation of learning found in both a similar ballistic movement task [17], as well as on a sequential pinching task testing both speed and accuracy [15,16], which hints at some level of generalisability across tasks. However, to date, no clear candidate mechanism(s) for the improvement in consolidation has been identified and it remains unknown whether improvements in these distinct learning paradigms share a similar mechanism or not. The present data are broadly consistent with the previous reports of offline effects of TDCS on learning, though they differ in that here TDCS impaired, rather than improved, the retention of learning.

One implication of the above is that electrode placement may also influence the behavioural outcomes of TDCS, perhaps by targeting different populations of neurones within and even outside of M1 (e.g. somatosensory cortex). A second implication is that current flow in cortical areas between, and not just under, the electrodes should be considered attempting to understand the mechanisms of any behavioural changes. This may seem a fairly obvious point, particularly given that current flow models and intra-cranial recordings have already shown the existence of substantial current flow in regions between the electrodes [9,34]. However, until recently there was little direct evidence of any physiological consequences [10,11,35]. Therefore, from a practical stand point, when selecting an electrode montage to stimulate a particular area of cortex we should perhaps think about both its position and orientation with respect to the applied electric field.

Similar conclusions regarding the importance of electrode positioning have been reached in studies of the effects of TDCS on MEPs [12] and neuromuscular fatigue [36]. The influence of electrode montage could in part reflect variation in the strength of the electric field at the targeted cortical region, because the strength is known to vary at different sites in between the electrodes [9,34,37,38] and the outcomes of TDCS on M1 excitability show non-linear relationships with the strength of the applied current [39] (though we note there is some uncertainty surrounding the precise shape of the dose-response relationship [40]). Alternatively, and in line with the above considerations, electrode montage might also influence the outcomes of TDCS by changing the orientation of current flow orientation with respect to the neural elements in the electric field, and consequently which elements are modulated.

### TDCS-independent effects of motor practice on retention

The transfer of motor memories from a relatively unstable state to a more stable state is thought to be enhanced by the repetition of movements at near-asymptotic levels of performance [29]. Consistent with this, we found a weak correlation between the slope of learning in the second half of practice and the retention of learning, which suggests that individual who spent more time practicing at close to peak levels of performance tended to maintain their current performance levels better than those who spent less time. This relationship was independent of the effect of TDCS on retention, as was clear from the fact that TDCS did not affect the amount or rate of learning, and implies the presence of two dissociable processes involved in the retention of motor learning.

### Dissociation between TDCS effects on learning/retention and corticospinal excitability

A common observation in human experiments of motor learning is an increase in corticospinal excitability after practice [[19], [20], [21],[41], [42], [43], [44]]. The increase is thought to reflect the strengthening of synaptic connections in M1 and play a fundamental role in the formation and retention of new motor memories [[19], [20], [21],[41], [42], [43], [44]]. We found a muscle-specific increase in MEPs in the task-relevant APB muscle following practice in the TDCS_Sham_ group, which taken at face value would seem consistent with the idea. If this were true, and the neural elements involved in behavioural learning/retention overlapped with those generating the MEP, then we would expect to see corresponding changes in behavioural learning/retention and corticospinal excitability as a result of directional TDCS. This, however, was not the case.

The complete dissociation of behavioural and physiological markers of learning and retention, evident in both the lack of relationship between them and the capability to modify them independently via TDCS, suggests that such ad hoc explanations for the increase in corticospinal excitability after motor-practice cannot be correct. This notion is consistent with the fact that, although some studies have reported correlations between changes in corticospinal excitability and performance improvements [19,41], the majority have not [21,[43], [44], [45], [46]]. Instead, the increase in excitability could reflect activity-dependent changes in excitability that are not directly related to learning or retention processes. For example, repetitive activation of excitatory inputs to hippocampal pyramidal cells has been shown to increase persistent sodium currents in the post-synaptic dendrites [47]. The changes are caused by activation of metabotropic glutamate receptor-mediated calcium signalling and lead to enhanced dendritic excitability. One could imagine a similar activity-dependent increase in corticospinal cell excitability that is blocked by TDCS, perhaps by altering calcium signalling.

Motor practice and combined motor practice and TDCS produced changes in MEPs that were consistent across both directions of TMS, implying that they were not specific to either the putative PA or AP input pathways and thus could reflect altered excitability of the corticospinal output neurones themselves. Behavioural learning and retention in this task could therefore potentially occur upstream of the corticospinal output neurones, for example in other pre-synaptic inputs not recruited by the TMS pulses or even in other areas of the cortex such as the somatosensory cortex. Alternatively, learning and retention may directly involve the corticospinal output neurones, but these changes may not be reflected well in the complex signal of the MEP since it contains cortico-cortical, intracortical and spinal contributions. In fact, improvements in ballistic motor performance with practice have been shown to coincide with facilitation at the spinal level [48]. Alterations in excitability at a spinal level (e.g. corticomotoneuronal synapse) could potentially explain the similar changes in PA and AP MEPs, though if this were the case it would be difficult to explain why TDCS applied to the cortex affected the modulation of MEPs after motor practice.

### Limitations

Effect sizes for the main analyses in the present study were generally large, suggestive of a seemingly meaningful interaction between directional TDCS and the physiological and behavioural outcomes of motor practice. However, whilst the sample size here is consistent with previous studies of TDCS effects on learning [[15], [16], [17]], it could be considered relatively modest. Effect sizes have the potential to be over-inflated for smaller sample sizes, and thus the present results should be considered preliminary. Further studies with larger sample sizes are required confirm the importance of TDCS current flow and electrode location on motor learning and the generalisability of these issues to other areas of cortex as well as other behaviours.

## Conclusions

The results are consistent with the idea that electric field direction is of relevance to the behavioural outcomes of TDCS. We speculate that lack of control over this parameter, as well as variation in the individual folding of the human cortex, could account for some of the variability previously observed in the physiological and behavioural effects of TDCS. The results also confirm the increase in corticospinal excitability after a period of motor practice is not causally related to behavioural learning or retention as is often assumed.

## Conflicts of interest

The authors report no conflicts of interest.

## Funding

RH and JCR were supported by a Biotechnology and Biological Sciences Research Council grant (BB/N016793/1).
