# Supplementary material for: Direction of TDCS current flow in human sensorimotor cortex influences behavioural learning
Source: Brain Stimul. 2019 May-Jun;12(3):684–92. doi: 10.1016/j.brs.2019.01.016 (PMC6491497; doi:10.1016/j.brs.2019.01.016)
Supplement: Multimedia component 1 [file mmc1.pdf]

Supplementary Figure 1.

Methods

We measured the magnitude of the resultant acceleration vector from the x- and z-axis (Vxz) [Equation 1: $Vxz = \sqrt{Vx^2 + Vz^2}$] and the angular direction of the resultant vector (θxz in degrees relative to the horizontal, where 0° is horizontal and therefore reflects movements purely in the x-axis) [Equation 2: $\theta = \tan^{-1}(Vx/Vz)$]. Vx is the area under the curve x-axis acceleration time curve from onset to peak acceleration, and Vz is the area under the curve z-axis acceleration time curve from onset to peak acceleration measured in the x-axis. The area was measured here, rather than simply the peak amplitude, since it would seem to better convey the mean direction of the movement than the amplitude at the instantaneous peak acceleration. The data were entered into an ANOVA with trial epoch (Basline, 1, 2…12) as a within-subjects factor and TDCS group ($TDCS_{PA}$, $TDCS_{AP}$ and $TDCS_{Sham}$) as a between-subjects factor.

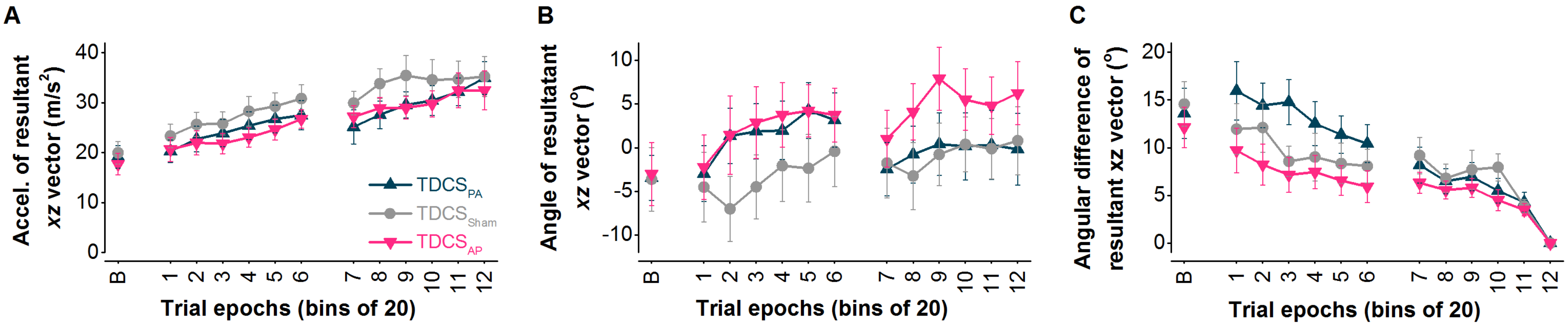

Supplementary figure 1. A. The resultant acceleration vector in the x- and z-axis (*Vxz*) increased similarly on day 1 for each of the three TDCS groups. **B.** The resultant angle of vector *Vxz* increased over the course of practice on day 1, with no differences between the TDCS groups. **C.** The absolute angular difference between the baseline epoch and the final epoch decreased over the course of practice, and reduction was similar for all three TDCS groups.

Results

Overall the magnitude of the resultant vector on day 1 followed a similar pattern as the peak acceleration in the x-axis as reported in the main text, increasing over the course of practice similarly in each of the three TDCS groups (S1A). This was confirmed by a significant main effect of trial epoch ($F_{[12,540]} = 30.946$, $P < 0.001$, $\eta p^2 = 0.407$), with no main different between TDCS conditions ($F_{[2,45]} = 0.737$, $P = 0.484$, $\eta p^2 = 0.032$) and no interaction of TDCS condition × trial epoch ($F_{[24,540]} = 30.946$, $P = 0.967$, $\eta p^2 = 0.023$).

As predicted, the data also showed a rotation in the angle of the resultant vector over the course of practice, but this was not affected by TDCS (S1B). Again this was confirmed by a significant main effect of trial epoch ($F_{[4.012,180.556]} = 3.22$, $P = 0.014$, $\eta p^2 = 0.067$), with no main different between TDCS conditions ($F_{[2,45]} = 0.789$, $P = 0.461$, $\eta p^2 = 0.034$) and no interaction of TDCS condition × trial epoch ($F_{[24,540]} = 0.923$, $P = 0.571$, $\eta p^2 = 0.039$). Since the angle of the resultant vector at the start and end of practice varied widely across individuals in sign and magnitude, we also calculated the absolute angular difference between the final epoch on day 1 and each of the previous practice epochs (baseline to epoch 11), to better illustrate the rotation in the angle with practice (S1C). ANOVA revealed a significant main effect of trial epoch ($F_{[3.997,178.969]} = 3.232$, $P = 0.014$, $\eta p^2 = 0.065$), with no main different between TDCS conditions ($F_{[2,45]} = 0.934$, $P = 0.400$, $\eta p^2 = 0.040$) and no interaction of TDCS condition × trial epoch ($F_{[22,495]} = 0.903$, $P = 0.591$, $\eta p^2 = 0.039$).
