# Supplementary material for: Direction of TDCS current flow in human sensorimotor cortex influences behavioural learning
Source: Brain Stimul. 2019 May-Jun;12(3):684–92. doi: 10.1016/j.brs.2019.01.016 (PMC6491497; doi:10.1016/j.brs.2019.01.016)
Supplement: Multimedia component 2 [file mmc2.pdf]

Supplementary Figure 2.

Methods

We quantified the average slope of the relationship between mean acceleration and practice epoch for each individual over the first and second blocks of practice (i.e. trials 1-120 and 121-240) on day 1. Data were from each epoch were first expressed as a percentage increase relative to the baseline block before calculating the slope. We expected that a shallower slope of learning in the second block of practice, indicative of lower rate of learning and presumably plateauing performance levels, would be negatively related to the retention of performance levels at the start of practice on day 2.

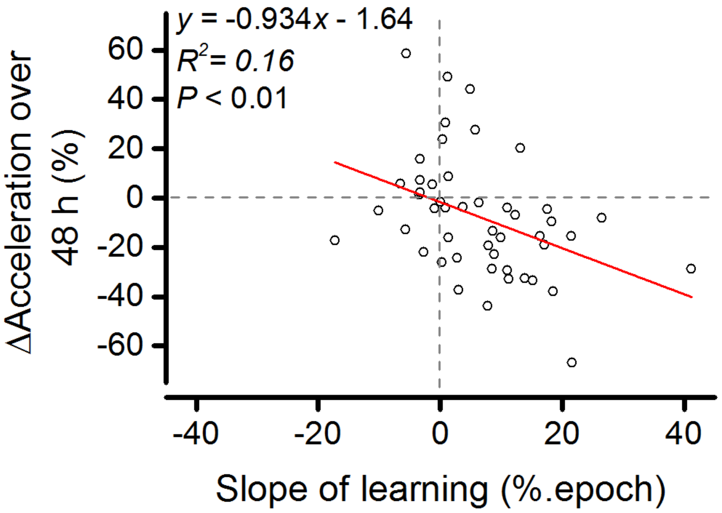

Results

First, ANOVA with the slope in each block (1 and 2) as a within-subjects factor and TDCS group ($TDCS_{Sham}$, $TDCS_{PA}$, $TDCS_{AP}$) as a between subjects factor, confirmed that there were no differences between the TDCS groups with respect to the slope in block 1 or 2. This is consistent with the lack of influence of TDCS on learning in the main text. There was no main effect of block ($F_{[1,45]} = 0.654$, $P = 0.423$, $\eta p^2 = 0.014$) or TDCS group ($F_{[2,45]} = 0.385$, $P = 0.683$, $\eta p^2 = 0.017$), and no interaction of block and TDCS group ($F_{[2,45]} = 0.347$, $P = 0.709$, $\eta p^2 = 0.015$). Partial correlation applied to the whole cohort ($n = 48$), and controlling for the effect of TDCS condition, then revealed a significant negative correlation between the retention and the rate of learning in block 2 ($r = -0.438$, $P = 0.002$). No relationship was observed with the rate of learning in the first block of practice ($r = -0.192$, $P = 0.096$). These results imply that individuals with a lower rate of learning in the second block of practice, i.e. those who were presumably close to asymptotic levels of performance, tended to maintain performance levels or even consolidate with further performance improvements over the intervening 48 h whilst those who were still learning at a high rate tended to experience a drop in performance.

To establish whether TDCS and the rate of learning in the second block of practice were independent contributors to retention of performance we entered the data into a step-wise linear regression with the change in acceleration over 48 h as the dependent variable and TDCS group, rate of learning in block 1 and 2, and the relative change in MEP amplitudes (averaged across TMS_{PA} and TMS_{AP}) as predictor variables. Only TDCS group and the rate of learning in block 2 were entered into the model, and they independently explained 9% and 16% of the variance in retention, respectively (total variance explained 25%; ANOVA, $F_{[2,45]} = 7.629$, P < 0.001).
